# Food and Drug Administration Approval Summary: Odevixibat (Bylvay) for the Treatment of Pruritus With Progressive Familial Intrahepatic Cholestasis

**DOI:** 10.1016/j.gastha.2024.100596

**Published:** 2024-11-29

**Authors:** Sojeong Yi, Insook Kim, Rebecca Hager, Marian M. Strazzeri, Lili Garrard, Toru Matsubayashi, Ruby Mehta

**Affiliations:** 1 Division of Inflammation and Immune Pharmacology, Office of Clinical Pharmacology, Office of Translational Sciences, Center for Drug Evaluation and Research (CDER), The U.S. Food and Drug Administration (FDA), Silver Spring, Maryland; 2 Division of Biostatistics III, Office of Biostatistics, Office of Translational Sciences, CDER, FDA, Silver Spring, Maryland; 3 Division of Hepatology and Nutrition, Office of Immunology and Inflammation, Office of New Drugs, CDER, FDA, Silver Spring, Maryland

**Keywords:** Clinical Outcome Assessment (COA), Pruritus, Itching, Progressive Familial Intrahepatic Cholestasis (PFIC), Adverse Events (AE)

## Abstract

On July 20, 2021, the Food and Drug Administration approved odevixibat (Bylvay) for the treatment of pruritus in patients 3 months of age and older with progressive familial intrahepatic cholestasis (PFIC). PFIC is a rare disease that results in impaired bile secretion and transport, leading to cholestatic liver injury. Odevixibat is a reversible inhibitor of the ileal bile acid transporter. It decreases the reabsorption of bile acids from the terminal ileum (distal small intestines). Approval was based on the improvement in pruritus demonstrated in a 24-week randomized double-blind placebo-controlled trial conducted in 62 pediatric subjects, aged 6 months to 17 years, with a confirmed molecular diagnosis of PFIC type 1 or type 2 with the presence of pruritus at baseline. Given the subjects’ young age, a single-item observer-reported outcome (ObsRO) was used to measure scratching as observed by the caregiver. Subjects had an average scratching score of greater than or equal to 2 (medium scratching) in the 2 weeks before baseline. The mean percentage of ObsRO assessments over the 24-week treatment period that were scored as 0 (no scratching) or 1 (a little scratching) was 35.4% and 30.1% for 40 mcg/kg/day and 120 mcg/kg/day odevixibat treatment, respectively, compared to 13.2% for placebo. There was general alignment between subject and caregiver assessments of worst weekly pruritus severity among subjects for whom both patient-reported outcome (Worst Weekly Itching Score) and ObsRO (Worst Weekly Scratching Score) data were available. The most common adverse reactions included diarrhea, liver test abnormalities, vomiting, abdominal pain, and fat-soluble vitamin deficiency. The benefit-risk assessment for odevixibat for the treatment of pruritus in the labeled population was considered favorable.

## Introduction

Progressive familial intrahepatic cholestasis (PFIC) is a rare, autosomal recessive disease that results in impaired bile secretion and transport, leading to cholestatic liver injury. PFIC are a heterogenous group of diseases that affects one in 50,000–100,000 births.^1^ PFIC1 and PFIC2, which result from mutations in the ATP8B1 and ABCB11 genes, respectively, are the most common types of PFIC, constituting about 2-thirds of cases. Additional gene mutations associated with PFIC have been identified and described in the literature.^2^^,^^3^

PFIC presents during early childhood with pruritus, elevated liver tests, jaundice, fat-soluble vitamin (FSV) deficiency, and growth failure.^3^ PFIC1 and PFIC2 present with elevated aspartate aminotransferase, alanine aminotransferase, total bilirubin, total serum bile acid (sBA), and direct bilirubin, and normal serum gamma glutamyl transpeptidase. PFIC can further progress to cirrhosis and liver failure, and up to 15% of patients with PFIC2 can develop hepatocellular carcinoma.^4^ Patients with PFIC1 can also present with extrahepatic symptoms, such as pancreatitis or deafness.^5^

Patients with PFIC can experience pruritus of varying severity. The physical manifestations of pruritus range from scratch marks to excoriations and scarring caused by persistent and unrelenting pruritus.^6^ Itching and scratching are disabling symptoms and can be disruptive for patients and their families, limiting the patient’s ability to participate in daily activities.^7^ Families of patients with PFIC shared their accounts of the impact of the disease at patient-led listening sessions conducted in 2018 and 2021 by Professional Affairs and Stakeholder Engagement at the FDA. Pruritus emerged as one of the most devastating symptoms that adversely affected the quality of life of both patients and caregivers.

Off-label drug treatments for pruritus include ursodeoxycholic acid (UDCA), cholestyramine, antihistamines, naltrexone, rifampin, and ondansetron.^8^^,^^9^ Most of these therapies do not ameliorate pruritus and some cause undesirable adverse events (AEs). Pruritus that is refractory to drug treatment is severe, affects quality of life, and may necessitate surgical biliary diversion or liver transplantation.^8^ Treatment of cholestatic pruritus was an unmet medical need at the time of FDA review of the application. Although several molecules (eg, bile acids, autotaxin, endogenous opioids) have been recognized as a potential cause for cholestatic pruritus, the pathophysiology of pruritus in PFIC is not completely understood.^10^^,^^11^

Odevixibat is an orally administered drug that inhibits the ileal bile acid transporter (IBAT) located in the apical membrane of the terminal ileum. IBAT uptakes bile acids in the salt form from the ileum as the initial step of enterohepatic circulation. By inhibiting IBAT, odevixibat decreases reabsorption of bile acids, subsequently increasing bile acid excretion through the colon. Odevixibat is designed to act locally in the gut lumen where it binds reversibly to IBAT showing minimal systemic absorption following oral administration.

On July 20, 2021, the FDA approved odevixibat (Bylvay) for the treatment of pruritus in patients 3 months of age and older with PFIC.^12^^,^^13^ Bylvay is taken orally in the morning with a meal. The approved dosage of Bylvay is 40 mcg/kg once daily, which may be increased up to 120 mcg/kg. Bylvay is available in 2 dosage forms, ie, capsule, and oral pellets. Bylvay capsules (400 mcg and 1200 mcg strengths) are intended for patients ≥19.5 kg, whereas Bylvay oral pellets (200 mcg and 600 mcg strengths) are intended for those <19.5 kg. Bylvay oral pellets are to be mixed with soft food or age-appropriate liquids (eg, breast milk, formula, or water).

Herein, the authors summarize the FDA’s assessment and basis for the approval of odevixibat for the treatment of pruritus in patients with PFIC.

## Clinical Trial Design and Efficacy Assessment

The efficacy and safety of odevixibat was primarily supported by a single adequate and well-controlled phase 3 trial (Trial 1, NCT03566238).^14^ The investigator’s analyses and interpretation of the data from Trial 1 have been previously published.^14^

Trial 1 (NCT03566238) was a multicenter, double-blind, randomized, placebo-controlled trial to evaluate the efficacy and safety of odevixibat compared to placebo. It consisted of a 35- to 56-day screening period, a 24-week treatment period, and a 4-week follow-up period. The study enrolled subjects between the ages of ≥6 months and ≤18 years with a genetically confirmed diagnosis of PFIC1 or PFIC2; history of significant pruritus; caregiver-reported observed scratching in electronic diaries average of ≥2 during the screening period; and elevated sBAs at baseline >100 μmol/L.

The protocol planned to enroll approximately 60 eligible subjects who were randomized in a 1:1:1 ratio to receive 40 mcg/kg/day of odevixibat, 120 mcg/kg/day of odevixibat, or placebo, for up to 24 weeks. Randomization was stratified by PFIC class (types 1 and 2) and age group (6 months to 5 years, 6–12 years, and 13 to ≤18 years).

After randomization, subjects were to return to the clinic at weeks 4, 8, 12, 18, 22, and 24/end of treatment for assessment. Concomitant treatment with conventional therapies, including UDCA and rifampicin, was permitted during the study, provided the dose remained stable during the treatment period. All randomized subjects, ie, the intent-to-treat population, were included in the efficacy and safety analyses.

### Clinical Outcome Assessments (COAs) of Pruritus to Evaluate Efficacy

Given the subjects’ young age, a single-item observer-reported outcome (ObsRO) measure of pruritus (scratching) severity was chosen as the appropriate primary clinical outcome assessment (COA) measure to support the evaluation of efficacy, as it could be administered to all subjects across the age range in the trial.^15^ This was the first-time that an ObsRO was used to evaluate efficacy of a drug product for PFIC.

Throughout the trial period, a single-item ObsRO measure of pruritus (scratching) severity was recorded twice daily via electronic diaries by the caregivers of all enrolled subjects—once in the morning (AM) and once in the evening (PM). Caregivers rated subjects’ worst nighttime scratching severity (since going to bed the night before) and worst daytime scratching severity (since waking up that morning) on a 5-point ordinal response scale with possible scores of 0 (no scratching), 1 (a little scratching), 2 (medium scratching), 3 (a lot of scratching), and 4 (worst possible scratching).

In addition to the ObsRO, subjects aged 8 years and older completed analogous single-item AM and PM patient-reported outcome (PRO) measures of pruritus (itching) severity according to the same schedule as the corresponding ObsRO measures. Subjects rated their worst nighttime and daytime itching severity, respectively, on a 5-point ordinal response scale with possible scores of 0 (no itching), 1 (a little itching), 2 (medium itching), 3 (a lot of itching), and 4 (the worst itching).

The applicant’s prespecified primary endpoint was based on the mean percentage of “positive pruritus assessments.” However, FDA had concerns about the applicant’s definition of a “positive pruritus assessment” as described below, and subsequently considered an alternative definition for the primary efficacy evaluation.

#### Applicant’s prespecified primary endpoint

The protocol prespecified the primary efficacy endpoint as the mean percentage of “positive pruritus assessments” that each subject experienced over the 24-week treatment period. A positive pruritus assessment was defined as (1) a daytime or nighttime scratching score of ≤1 or (2) a decrease in daytime or nighttime scratching score of 1 point or more from the baseline average. Baseline average daytime and nighttime scratching scores were computed as the arithmetic mean of the daily daytime and nighttime scratching scores, respectively, over the 14 consecutive days immediately before the first dose of study medication, and then rounded to the nearest integer.

To determine whether a pruritus assessment represented a decrease in daytime or nighttime scratching score of 1 point or more from the baseline average (ie, met the second criterion for a positive pruritus assessment), each (daily) daytime scratching score was compared to the (2-week) baseline average daytime scratching score, and each (nightly) nighttime scratching score was compared to the (2-week) baseline average nighttime scratching score. The mean value of the percentage of positive pruritus assessments for each subject was compared across study arms, ie, the mean percentage of assessments that were ≤1 or at least a 1 point decrease from baseline.

FDA had concerns about the second criterion (a decrease in daytime or nighttime scratching score of 1-point or more from baseline) in the Applicant’s prespecified definition of a positive pruritus assessment. First, this criterion involved comparing a subject’s daily daytime and nighttime scratching severity during treatment with their baseline average daytime and average nighttime scratching severity, respectively, over 14 consecutive days. Aggregating COA data over different lengths of time (ie, each day vs 14 days at baseline) yields different clinical outcomes, and in Trial 1, these comparisons were insurmountably challenging to interpret. Second, comparing an integer that can take a value of 0, 1, 2, 3, or 4 to an average that can take any real number value between 0 and 4, inclusive, (eg, 3.6) is challenging. Although rounding the baseline average should not affect the treatment arms in a differential manner, it was not clear whether a summary measure that is impacted by rounding constitutes a meaningful summary of outcomes. Third, the clinical meaningfulness of a decrease in daytime or nighttime scratching score of 1 point or more from baseline was insufficiently justified.

#### FDA’s alternative primary endpoint

Given the limitations of the prespecified primary endpoint, FDA alternatively defined a positive pruritus assessment as an ObsRO response of either no scratching (scratching score = 0) or a little scratching (scratching score = 1) as this alternative definition was considered a more interpretable and inherently meaningful criterion of clinical benefit. FDA evaluated an alternative primary efficacy endpoint in which a positive pruritus assessment was defined solely as a daytime or nighttime scratching score of ≤1. This alternative definition omitted the second criterion from the original definition of a positive pruritus assessment comparing a daily score to a 2-week baseline average. The mean value of the percentage of positive pruritus assessments in each subject was compared across study arms, ie, the mean percentage of assessments ≤1.

#### Statistical analysis of primary endpoint

For both the Applicant’s prespecified primary endpoint and the FDA’s alternative primary endpoint, the subject-level percentages of positive pruritus assessments over the 24-week treatment period were evaluated using a prespecified analysis of covariance model, which included variables for treatment arm, baseline average daytime pruritus score, baseline average nighttime pruritus score, and the randomization stratification factors, i.e., PFIC type and age group. The Applicant prespecified that all missing scratching assessments after intercurrent events (ie, premature treatment discontinuation, death, or initiation of rescue treatments) were considered negative pruritus assessments (ie, not meeting the criteria of a positive pruritus assessment).

A multiple testing procedure was prespecified to control the type I error rate for evaluating the primary efficacy endpoint for each of the 2 dose groups compared to the placebo group. In the closed testing procedure, the average effect of the 40 mcg/kg/day and 120 mcg/kg/day dose groups were first compared with the placebo group. If the 1-sided *P* value was ≤0.025, the 1-sided *P* values were calculated for the comparisons of the 40 mcg/kg/day dose vs placebo and the 120 mcg/kg/day dose vs placebo. A significant treatment effect was declared for the dose group(s) with a *P* value ≤.025. No adjustments were specified for multiple comparisons when testing secondary and exploratory efficacy variables.

#### Additional pruritus evaluations

FDA additionally analyzed an efficacy endpoint not included in the testing hierarchy: change in Worst Weekly Scratching Score (WWSS) from baseline to Month 6 (weeks 21–24). FDA developed the WWSS by evaluating the comparability of daytime and nighttime scratching scores within a given day and the stability of daytime and nighttime scratching scores over time. Based on the results of FDA’s analyses revealing notable within- and between-day variability in scratching scores, FDA computed a subject’s WWSS over a 4-week (28-day) assessment period as described next. First, a subject’s Worst Daily Scratching Score was computed as the highest (worst) of the daytime and nighttime scratching scores on a given day and was considered missing if both the daytime and nighttime scratching scores for that day were missing. Second, the average of the Worst Daily Scratching Scores across the 7 days in a week was computed for each of the 4 weeks in the assessment period and was considered missing for a given week if more than 3 of the Worst Daily Scratching Scores were missing. Third and finally, the WWSS was computed as the highest (worst) of these weekly averages and was considered missing if all 4 weekly averages were missing. Missing data were not imputed in the computation of a subject’s WWSS.

The endpoint mean change in WWSS from baseline to month 6 (weeks 21–24) was evaluated using a mixed-effect model for repeated measures including baseline pruritus score, treatment group, month, treatment-by-baseline pruritus score interaction, treatment-by-month interaction, and stratification factors (PFIC type and age group) with an unstructured covariance matrix to compare treatment effects over the last 4 weeks of blinded treatment (i.e., Month 6). This analysis model was chosen by FDA because it was prespecified by the Applicant for other exploratory endpoints.

**Figure A1:**
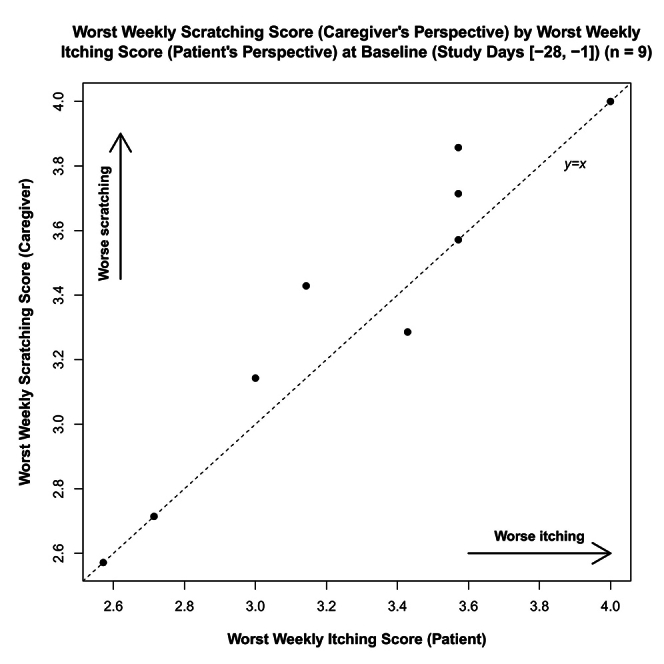
Comparability of Patient and Caregiver Pruritus Assessments. In FDA’s analyses, patients were pooled across trial arms and missing data were not imputed.

As a subject’s itching severity is known only to the subject, FDA considered it important to understand the relationship (comparability) between itching severity as directly reported by subjects and scratching severity as observed by caregivers. To this end, FDA conducted post hoc descriptive analyses to compare Worst Weekly Itching Scores (PRO-based) and WWSS’s (ObsRO-based) among 9 subjects for whom there were both PRO and ObsRO score data available during the 4 weeks before randomization. These analyses suggested general alignment between subject and caregiver assessments of worst weekly pruritus severity (Figure A1). Additional analyses conducted by FDA (results not shown) also indicated general alignment between daily subject and caregiver assessments of daytime and nighttime pruritus severity with strong alignment in the worst daily pruritus scores.

### Change from Baseline in sBA

Total sBA levels were measured at the following time points: screening period (day 56, day 28), at baseline (at randomization, day 0), and weeks 4, 8, 12, 18, 22, and 24. The protocol prespecified sBA reduction as a secondary efficacy endpoint, ie, the proportion of subjects with at least a 70% reduction in sBA from baseline or reaching a level ≤70 μM after 24 weeks of treatment, in the absence of multiplicity adjustment specified to test secondary endpoints.

However, FDA considered the sBA reduction as an exploratory endpoint and a pharmacodynamic biomarker reflecting the proposed mechanism of action of odevixibat. While sBA has been suggested as a pruritogen, the exact cause of pruritus in PFIC is not fully understood and cholestatic pruritus is considered multifactorial. The relationship between sBA, or the reduction of sBA and the severity or improvement in pruritus, has not been well established. Therefore, the clinical meaningfulness of the cutoff values of sBA reduction (ie, 70% reduction from baseline or reaching ≤70 μM) was unclear.

Further, due to the lack of reliability of the reported sBA values, FDA did not use quantitative comparisons of sBA as prespecified (eg, 70% change from baseline) and interpreted sBA levels only in a qualitative manner based on the overall trend. The sBA was analyzed using a commercial kit in Clinical Laboratory Improvement Amendments-certified laboratories. However, the bioanalytical method validation parameters for sBA levels used in the trial did not meet the validation criteria to support the quantitative assessment of response to treatment per the FDA bioanalytical guidance.^16^ For instance, 54% of the reported sBA values exceeded the upper limit of quantitation (ie, >180 μM) of the commercial kit. The serum samples exceeding >180 μM were diluted to lower the concentrations to be within the quantitation range. However, the dilution linearity was not established to support the reliability of sBA concentrations measured after sample dilution.

## Results

### Patient Demographic (Safety and Efficacy Analysis Population)

Characteristics of the intent-to-treat population (N = 62) are summarized in Table 1. The median age of the subjects was 3.2 years and ranged from 6 months to 15.9 years. Most subjects (n = 47, 76%) were between 6 months and 5 years of age. A majority of subjects (n = 45, 73%) had PFIC2 and 17 (27%) had PFIC1. The median time since diagnosis of PFIC was 1.5 years. The majority of subjects (n = 55, 89%) were receiving UDCA and/or rifampicin at study entry, with 50 subjects (81%) on UDCA and 41 (66%) on rifampicin.

**Table 1:** Baseline Demographic and Clinical Characteristics, ITT Population, Trial 1

Characteristic	Placebo N = 20	Odevixibat 40 mcg/kg/day N = 23	Odevixibat 120 mcg/kg/day N = 19
Sex, n (%)			
Male	12 (60)	11 (48)	8 (42)
Female	8 (40)	12 (52)	11 (58)
Age, y			
Mean (SD)	3.7 (3.9)	3.9 (3.7)	5.2 (4.2)
Median (min, max)	2.8 (0.5, 15.0)	3.2 (0.6, 15.9)	4.9 (1.0, 13.2)
Age groups (y), n (%)			
6 mo to 5 y	16 (80)	17 (74)	14 (74)
6–12 y	3 (15)	5 (22)	4 (21)
13–18 y	1 (5)	1 (4)	1 (5)
Race, n (%)			
White^a^	17 (85)	18 (78)	17 (89)
Asian	1 (5)	0 (0)	1 (5)
Black/African American	0 (0)	2 (9)	0 (0)
Other	2 (10)	3 (13)	1 (5)
Region of participation n (%)			
United States	3 (15)	2 (9)	3 (16)
Europe	12 (60)	13 (57)	10 (53)
Rest of world	5 (25)	8 (35)	6 (32)
PFIC type, n (%)			
Type 1	5 (25)	7 (30)	5 (26)
Type 2	15 (75)	16 (70)	14 (74)
AM baseline pruritus score			
Mean (SD)	3.0 (0.2)	3.1 (0.1)	2.9 (0.1)
Median (min, max)	3.0 (1.5, 4.0)	3.1 (2.1, 4.0)	2.9 (2.0, 3.5)
PM baseline pruritus score			
Mean (SD)	2.9 (0.1)	2.9 (0.1)	2.6 (0.1)
Median (min, max)	3.0 (1.7, 4.0)	2.9 (1.9, 4.0)	2.8 (1.1, 3.5)
Average of AM and PM baseline pruritus			
Mean (SD)	3.0 (0.1)	3.0 (0.1)	2.7 (0.1)
Median (min, max)	3.0 (1.9, 4.0)	3.0 (2.0, 4.0)	2.9 (1.6, 3.4)

AM, morning; ITT, intent to treat; max, maximum; min, minimum; N, number of subjects; n, number of subjects with at least 1 event; PFIC, progressive familial intrahepatic cholestasis; PM, evening; SD, standard deviation.

a The trial population from Middle Eastern countries was included under race category “White” by the Applicant.

### Efficacy for Pruritus

Table 2 summarizes the efficacy results for FDA’s alternative primary end point, the applicant’s prespecified primary end point, and the FDA’s additional pruritus end point, as discussed in Section 2. Based on the applicant’s prespecified primary endpoint, both the 40 mcg/kg/day and 120 mcg/kg/day doses of odevixibat demonstrated superiority over placebo (1-sided *P* values <0.025 for an average effect of the dose groups [*P* = .0019] and each dose group individually [*P* = .0016 and .0163, respectively]). For FDA’s alternative primary end point, the 40 mcg/kg/day dose had a nominal 1-sided *P* value less than 0.025, but the 120 mcg/kg/day dose had a nominal 1-sided *P* value greater than 0.025. For both versions of the primary end point, both the 40 and 120 mcg/kg/day odevixibat groups showed a numerically greater response compared to placebo. Both the 40 and 120 mcg/kg/day odevixibat groups showed numerically greater decreases in least squares mean WWSS compared to the placebo group.

**Table 2:** Efficacy Results over 24-wk Treatment Period in Trial 1 (ITT Population)

Results	Placebo N = 20	Odevixibat 40 mcg/kg/day N = 23	Odevixibat 120 mcg/kg/day N = 19
FDA alternative primary endpoint: Mean percentage of assessments that are ≤1			
LS mean (SE)	13.2 (8.7)	35.4 (8.1)	30.1 (9.0)
LS mean difference (95% CI)^a^		22.2 (4.7, 39.6)	16.9 (−2.0, 35.7)
Applicant’s prespecified primary endpoint: Mean percentage of assessments that are ≤1 or at least a 1-point decrease from baseline			
LS mean (SE)	30.1 (9.1)	58.3 (8.6)	51.8 (9.5)
LS mean difference (95% CI)^a^		28.2 (9.8, 46.6)	21.7 (1.9, 41.5)
FDA additional pruritus endpoint: Mean change in WWSS from baseline to mo 6 (wk 21–24)			
Baseline mean (SE)	3.4 (0.1)	3.4 (0.1)	3.2 (0.1)
Change from baseline at mo 6, LS mean (SE)^2^	−0.3 (0.3)	−1.1 (0.3)	−1.1 (0.3)
Change from baseline at mo 6, LS mean difference, (95% CI)^b^		−0.7 (−1.4, 0.0)	−0.8 (−1.5, −0.1)

Source: FDA’s Analysis.

CI, confidence interval; ITT, intent to treat; LS, least squares; SE, standard error.

a Difference represents odevixibat – placebo.

b No imputation of missing data before mixed-effect model for repeated measures, analysis (missing at random assumption).

**Figure 1:**
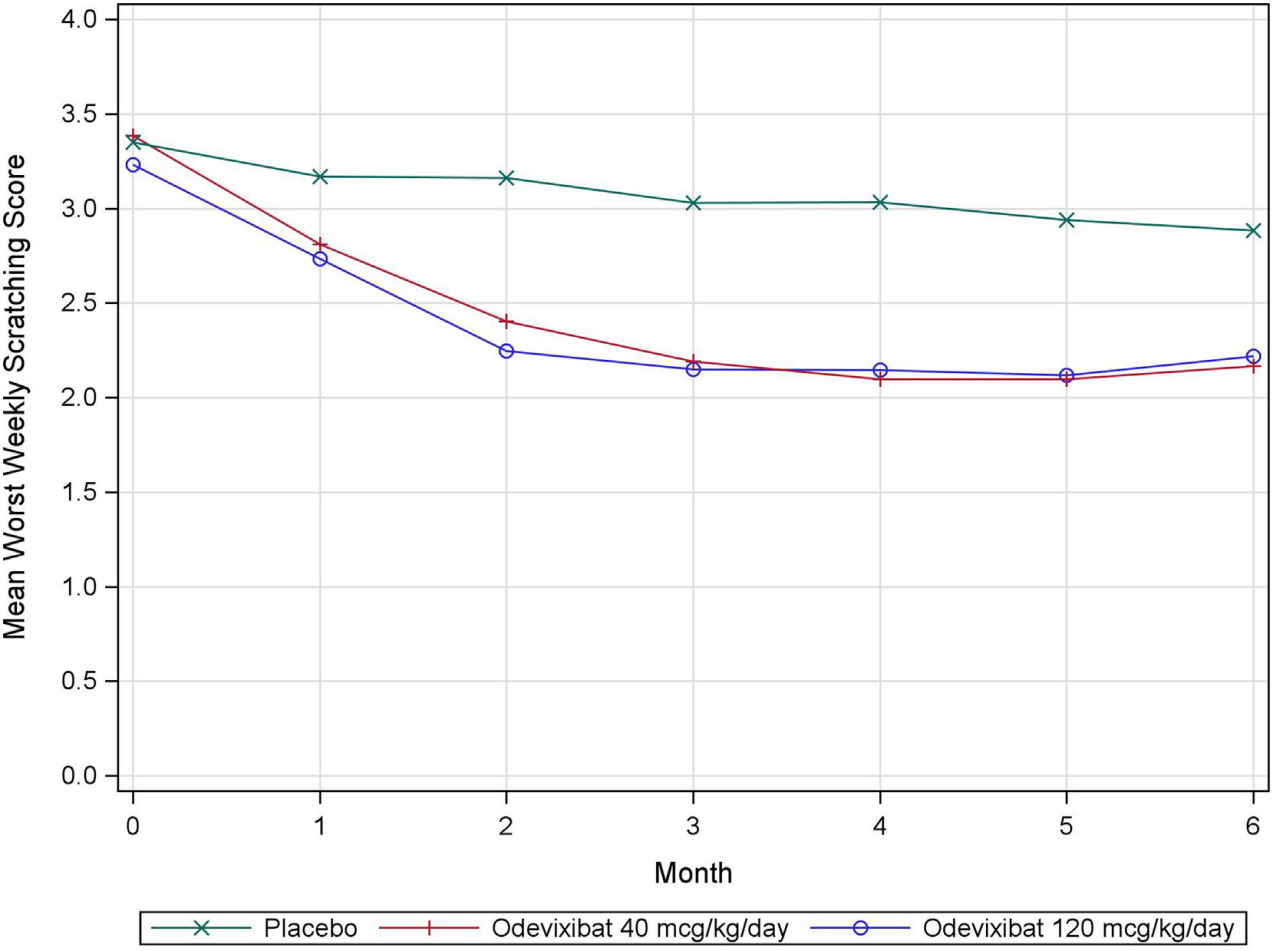
LS mean of WWSS’s in each month (ITT population).^1^^,^^2^ Source: FDA’s analysis.^1^ The analysis was based on a mixed model for repeated measures with baseline score as a covariate, and treatment group, time (in months), treatment-by-time interaction, treatment-by-baseline interaction, and stratification factors (PFIC type and age category) as fixed effects.^2^ A placebo-based multiple imputation approach under a missing not at random assumption was conducted as prespecified.

Figure 1 shows the least squares mean of the WWSS for each month. The odevixibat arms (40 and 120 mcg/kg/day) had similar mean levels of pruritus severity over the 6-month treatment period with clear separation from the placebo arm over time.

### Change from Baseline in sBA

**Figure 2:**
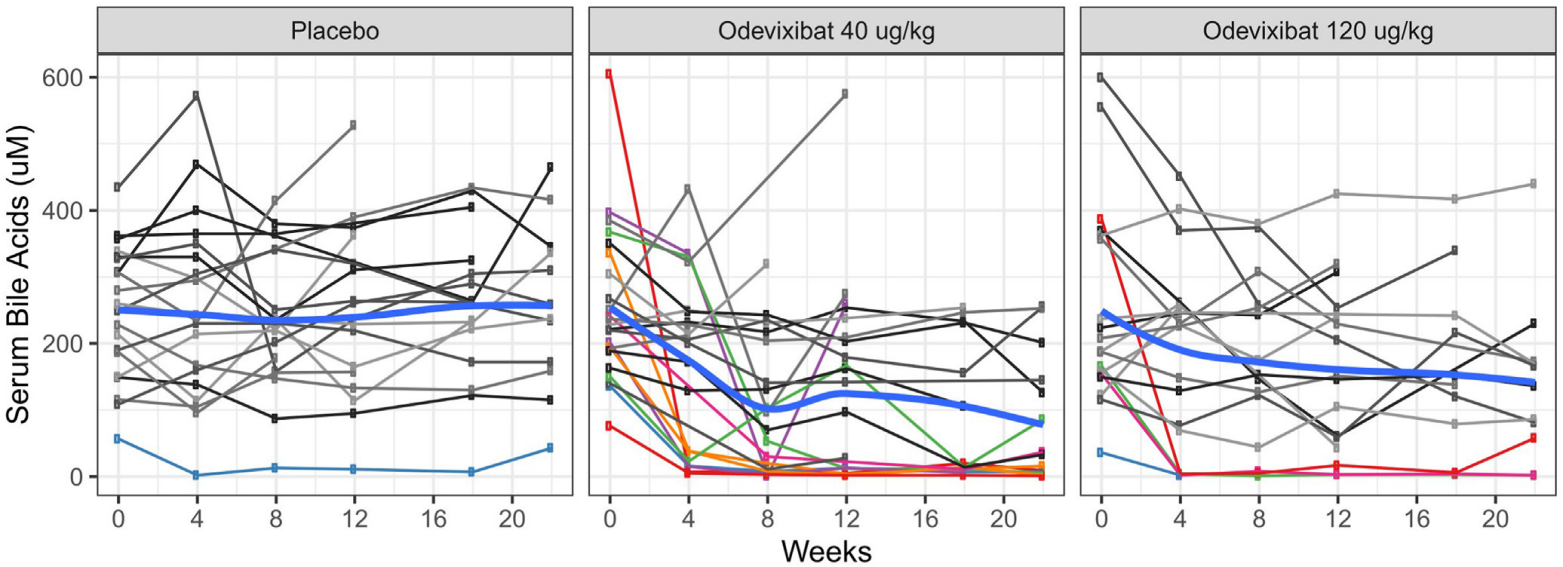
sBA levels over time in individual patients. Source: FDA’s analysis. Each line represents each individual patient. Colored lines represent subjects who achieved sBA levels below 10 μM (the upper limit of the normal range) 1 or more times during the treatment period. Bold blue lines represent locally estimated scatterplot smoothing regression.

In Trial 1, a decrease in sBA concentrations from baseline in subjects treated with odevixibat was observed within 4–8 weeks with variability among subjects, while sBA concentrations were generally not changed from baseline in subjects treated with placebo (Figure 2). The reduction of sBA concentrations from baseline appeared similar for both dose groups of odevixibat. Reduction in sBA concentrations below 10 μM (the upper limit of the normal range) at least once during the 24-week treatment period were achieved in 14 out of 42 (33.3%) odevixibat-treated subjects at either the 40 or 120 mcg/kg/day dose; and in 1 out of 20 (5%) placebo-treated-subjects (Figure 2, individuals with colored lines). Of the 15 subjects who had sBA below 10 μM at least once, 6 odevixibat-treated subjects were taking UDCA concomitantly and the placebo-treated subject was not on concomitant UDCA. Once sBA concentrations decreased, many odevixibat-treated subjects maintained a lower sBA concentration compared to baseline.

**Figure A2:**
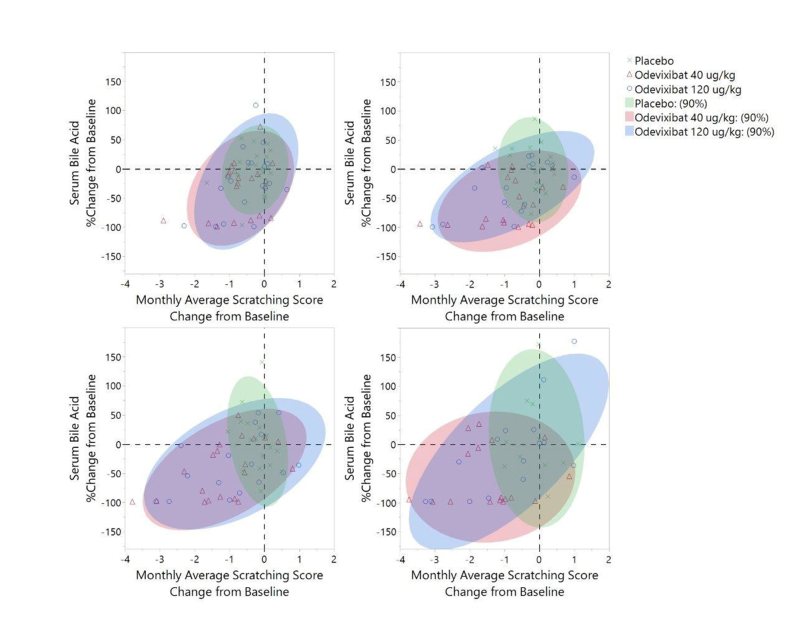
Percent Change From Baseline in Serum Bile Acid Levels Versus Change From Baseline in Monthly Average Scratching Scores by Time Period, Trial 1. Each area covers 90% of the data. “Y” axis represent change in sBA levels (horizontal dashed line denotes no change from baseline); “X” axis represents change in monthly averaged AM/PM ObsRO (scratch score), as pre-specified (vertical dashed line denotes no change from baseline).

In an exploratory analysis, a subset of odevixibat-treated subjects experienced both reduction in sBA levels and an improvement in pruritus (ie, ≥ 1-point decrease) (Figure A2).

### Safety

The safety database for the approval of odevixibat consisted of 62 subjects. The overall safety database in the premarketing trial was small; however, because PFIC is a rare disease, this was considered acceptable. Table 3 summarizes the frequency of clinical AEs, reported in ≥2% of subjects, and at a rate greater than in placebo subjects enrolled in Trial 1. The most common adverse reactions observed included diarrhea, liver test abnormalities, vomiting, abdominal pain, and FSV deficiency. Based on the mechanism of action of the drug, in the preclinical studies, and early phase clinical studies, FDA identified liver test abnormalities, diarrhea, and FSV deficiency as AEs of special interest potentially related to odevixibat treatment.

**Table 3:** AEs in Trial 1 Reported in ≥2% of ITT Population and at a Greater Rate Than in Placebo Group

Preferred term	Placebo N = 20 n (%)	Odevixibat 40 mcg/kg/day N = 23 n (%)	Odevixibat 120 mcg/kg/day N = 19 n (%)
Any AE	17 (85.0)	19 (82.6)	16 (84.2)
Diarrhea	2 (10.0)	9 (39.1)	4 (21.1)
AST or ALT increase	1 (5.0)	3 (13.0)	4 (21.1)
Vomiting	0	4 (17.4)	3 (15.8)
Abdominal pain	0	3 (13.0)	3 (15.8)
Blood bilirubin increased	2 (10.0)	3 (13.0)	2 (10.5)
Fat-soluble vitamin deficiency (A, D, E)	1 (5.0)	0	3 (15.8)
Splenomegaly	0	0	2 (10.5)
Cholelithiasis	0	0	1 (5.3)
Dehydration	0	0	1 (5.3)
Fracture	0	1 (4.3)	0
Liver tests elevations			
ALT increase over baseline by ≥ 150 U/L	0	2 (8.7)	2 (10.5)
AST increase over baseline by ≥ 150 U/L	0	1 (4.3)	3 (15.8)
TB increase over baseline by ≥ 2 mg/dL	1 (5.0)	4 (17.4)	1 (5.3)
DB increase over baseline by ≥ 1 mg/dL	2 (10.0)	5 (21.7)	2 (10.5)

ALT, alanine aminotransferase; AST, aspartate aminotransferase; DB, direct bilirubin; ITT, intent to treat; TB, total bilirubin; U/L, units per liter.

Most subjects enrolled in Trial 1 presented with abnormal liver tests at baseline. Elevations in alanine aminotransferase, aspartate aminotransferase, total bilirubin, and direct bilirubin were observed during the trial. These elevations occurred in a greater percentage of odevixibat-treated subjects compared to placebo-treated subjects. Treatment interruptions due to liver test abnormalities occurred in a higher percentage of odevixibat-treated subjects (7 of 42, 16.6%; 2 subjects receiving 40 mcg/kg/day and 5 subjects receiving 120 mcg/kg/day) subjects relative to placebo-treated subjects (2 of 20, 10%); however, none of the subjects permanently discontinued treatment due to liver test abnormalities. Treatment interruption and dose reduction of odevixibat may be warranted depending on the severity of the liver test abnormalities.

Diarrhea was reported in 2 (10%) placebo-treated subjects, 9 (39%) 40 mcg/kg/day odevixibat-treated subjects, and 4 (21%) 120 mcg/kg/day odevixibat-treated subjects. Treatment interruption due to diarrhea occurred in 2 subjects during treatment with 120 mcg/kg/day odevixibat and ranged from 3 to 7 days in duration. One patient treated with 120 mcg/kg/day odevixibat withdrew from the clinical trial because of persistent diarrhea. Interruption of odevixibat may need to be considered depending on the severity or duration of diarrhea. If diarrhea resolves, odevixibat can be restarted at 40 mcg/kg/day and increased as tolerated.

Patients with PFIC have FSV deficiency at baseline, and the absorption of FSV may be affected by odevixibat. New onset or worsening of existing FSV deficiency was reported in 2 (10%) placebo-treated subjects, 1(4.3%) in the 40 mcg/kg/day odevixibat-treated subjects, and 5 (26.3%) 120 mcg/kg/day odevixibat-treated subjects. However, ascertaining a causal relationship of FSV deficiency with odevixibat was not possible because of multiple confounding factors including the small number of subjects who developed FSV deficiency during the clinical trial, the short duration of the trial, and that all subjects were taking FSV supplementation (oral, intramuscular, intravenous routes etc.). To mitigate the potential risk of FSV deficiency, FSV levels should be obtained at baseline and monitored during odevixibat treatment. If FSV deficiency is persistent despite adequate FSV supplementation, the product label recommends odevixibat interruption or discontinuation.

## Regulatory Decision

### Benefit–Risk Assessment

Efficacy of odevixibat was demonstrated in Trial 1 on a direct measurement of clinical benefit for treatment of pruritus in patients with PFIC1 and PFIC2. Pruritus improvement for odevixibat-treated subjects compared to placebo subjects was observed on both the applicant’s prespecified primary endpoint and the FDA alternative primary endpoint (mean percentage of ObsRO assessments ≤1). FDA’s anchor-based analysis evaluating meaningful within-patient change in WWSS from baseline to month 6 suggested that odevixibat yielded meaningful within-patient improvement in scratching severity relative to placebo.

The mechanistic rationale for treatment of pruritus with odevixibat is supported by reduction of sBA level following administration of odevixibat observed in Trial 1, a proof-of-concept nonclinical study, and the phase 2 trial in pediatric subjects with cholestatic diseases. A reduction of sBA as a pharmacodynamic biomarker provided supportive evidence of the drug’s effect.

While odevixibat showed a clinical benefit on pruritus, the long-term benefits, eg, reduction of hepatic decompensation events or survival with native liver, have not been established and remain unknown. Trial 1 was not designed to demonstrate the efficacy of odevixibat in delaying long-term liver outcomes, such as reduction of hepatic decompensation events or survival with native liver. There are uncertainties about the relationship of sBA reduction and long-term clinical benefits, such as that sBA may not accurately reflect hepatocellular bile acids levels, which are the key mediators of liver damage and liver-related outcomes.

The safety information supports a favorable benefit-risk assessment. Up to 120 mcg/kg/day odevixibat showed negligible systemic exposure. Elevation in liver enzymes, diarrhea, and fat-soluble vitamin deficiency were identified as AEs potentially related to odevixibat. These AEs are monitorable and can be addressed by treatment interruption or discontinuation. These observations are included in the Warnings and Precautions (in the “Highlights of Prescribing Information” and “Section 5”) of the label^13^ to alert clinicians to carefully follow subjects who experience these AEs.

Because Trial 1 provided a safety database of fewer than 100 cases receiving odevixibat for ≤ 6 months, further safety evaluations will be continued postmarketing. An ongoing open-label, long-term extension trial (Trial A4250-008, NCT03659916) is planned to enroll 120 subjects with PFIC and follow them for at least 72 weeks to monitor for unusual safety issues that may arise with chronic use of odevixibat.

Moreover, malformations of the heart and great vessels in fetuses were reported in a nonclinical reproduction study in rabbits, and these occurrences remain unexplained given the limited systemic absorption of odevixibat. Since there are no available data on odevixibat use in pregnant women, FDA requires a postmarketing pregnancy registry.

### Indication Statement

FDA approved odevixibat for the treatment of pruritus in patients with PFIC aged 3 months and older.

The efficacy and safety of odevixibat has neither been established in patients with clinically significant portal hypertension nor in patients with decompensated cirrhosis. Therefore, FDA currently recommends that odevixibat be discontinued if patients experience a hepatic decompensation event.

#### PFIC subtypes

Although only PFIC1 and PFIC2 subtypes were evaluated in Trial 1, the indication statement includes all PFIC subtypes except for PFIC2/bile salt export pump protein-3 (BSEP-3). ABCC11 mutations in PFIC2/BSEP-3 result in nonfunctional or complete absence of BSEP which leads to low levels of bile salts in the intestine for reabsorption. Odevixibat may be ineffective when impaired intestinal bile acid excretion exists due to nonfunctional BSEP.

PFIC subtypes other than PFIC1 and PFIC2 are even rarer and conducting trials in these populations would be impracticable. Phenotypically, patients with all PFIC subtypes consistently present with cholestatic pruritus in a similar manner. Because odevixibat does not target specific genes or mutations, the efficacy of odevixibat for pruritus was extrapolated to all PFIC subtypes except for PFIC2/BSEP-3 and the labeling indication was approved for all PFIC subtypes.

#### Age

Although children 6 months of age and older were evaluated in Trial 1; the indication statement allows use in patients 3 months of age and older. PFIC can be diagnosed in infants 3 months of age or younger. Published data support that children as young as 3 months of age present with scratching or more commonly with irritability, as the motor skills in infants younger than 6 months are not developed.^17^ Multiple factors were considered while determining the age for which odevixibat was approved, ie, 3 months and older. Firstly, data in young infants, ie, from 6 months to 12 months of age, were submitted by the applicant, and from an ontogeny perspective, infants 3–6 months of age with PFIC are expected to respond similarly to infants 6–12 months of age.^18^ Secondly, the pathophysiology of the disease, ie, severe pruritus, has been reported in infants 3 months and older. There is an unmet medical need to treat pruritus in this population. Thirdly, the pharmacologic action of IBAT inhibition (decreased enterohepatic recirculation of bile acids) is expected to be maintained in younger infants. Finally, the drug is minimally absorbed, and AEs are monitorable, safety concerns in infants 3 to 6 months of age due to potential high drug exposure can be mitigated. The lower bound of the age limit of odevixibat approval down to age 3 months and older was to allow odevixibat access to younger infants with intractable pruritus.

### Dose and Dose Modification

The recommended dosage is 40 mcg/kg once daily. In Trial 1, no major efficacy difference between 40 and 120 mg/kg/day doses were observed for pruritus outcomes and reduction of sBA levels. Both 40 and 120 mcg/kg/day dosages had acceptable safety and benefit-risk profiles to support approval, although a higher percentage of subjects in the 120 mcg/kg/day odevixibat group had dose reductions or dose interruptions due to AEs (including diarrhea and liver test abnormalities) compared to those in the 40 mcg/kg/day odevixibat group (31.6% vs 13%, respectively).

As some subjects may benefit from a dose higher than 40 mcg/kg/day, a stepwise dose increase up to 120 mcg/kg/day in an increment of 40 mcg/kg/day may be considered for subjects who do not show improvement in pruritus within 3 months after starting odevixibat treatment at 40 mcg/kg/day.

## Conclusions

Odevixibat is the first product approved for the treatment of pruritus in patients 3 months of age and older for all types of PFIC except for a subtype of PFIC2 with BSEP-3. The approval is based on the clinically meaningful improvement of pruritus (based on an ObsRO) and acceptable safety profile after treatment with odevixibat. The reduction of sBAs in subjects with PFIC receiving odevixibat supported the mechanism of action and pharmacodynamic effects of odevixibat. At the time of drafting this manuscript, there was another product (maralixibat) in the same class approved for a similar indication.
